# Irak-4 rs4251481 gene variant: as a risk factor on inflammatory bowel disease

**DOI:** 10.3906/sag-1807-279

**Published:** 2019-04-18

**Authors:** Gonca CANDAN, Resul KAHRAMAN, Arzu ERGEN

**Affiliations:** 1 Department of Molecular Medicine, Aziz Sancar Institute of Experimental Medicine, İstanbul University, İstanbul Turkey; 2 Department of Gastroenterology, Ümraniye Education and Training Hospital, İstanbul Turkey

**Keywords:** Inflammation, inflammatory bowel disease, polymorphism, IRAK-4

## Abstract

**Background/aim:**

Abnormal immune response occurs in individuals who have alleles associated with innate and adaptive immune mechanisms that predispose to inflammatory bowel disease (IBD). Interleukin-1 receptor-associated kinase 4 (IRAK-4) involved in the pathway produces cytokines that initiate and maintain inflammation through Toll-like receptors and interleukin-1 receptors on the membranes of innate immune cells are stimulated with antigens. It was aimed to investigate whether IRAK-4 rs3794262 and rs4251481 polymorphisms predispose to IBD and the possible effects of these polymorphisms by examining these gene polymorphisms with the clinic and prognostic parameters of IBD.

**Material and methods:**

Real-time PCR technique was used to detect IRAK-4 polymorphisms in 107 patients with IBD and 103 healthy controls.

**Results:**

As a result of experimental studies, the frequency of occurrence of rs4251481 polymorphism related AG genotype (P = 0.029) and G allele (P = 0.005) was found to increase statistically in patients compared to controls. In the control group, the rs4251481 AA genotype rate of incidence increased compared with the patient group (P = 0.005).

**Conclusion:**

Consequently, this is the first study in terms of both polymorphisms on IBD. These results suggest that rs4251481 AG genotype and G allele are associated with increased IBD risk in patients.

## 1. Introduction

Inflammatory bowel disease (IBD) is a chronic autoinflammatory disease that affects the gastrointestinal tract. It is characterized by abdominal pain, weight loss, immune cell activation, inflammation, and ulceration (1). IBD includes Crohn’s disease (CD), ulcerative colitis (UC), and other conditions (2,3). These two major forms are characterized by similar patterns although they are distinct illnesses. UC presents with diffuse mucosal inflammation involving the rectum and adjacent colonic tissue, and CD may involve any part of the gastrointestinal tract, from the mouth to the anus. The etiology of IBD involves many factors such as genetic inheritance and microbiota (4). Innate and adaptive immunity-related single nucleotide polymorphisms (SNPs) are important in the predisposal to IBD (5).

Interleukin-1 receptor-associated kinase 4 (IRAK-4) is one of the elements of the toll-like receptor (TLR) signal pathway, which plays an important role especially in innate immunity. The IRAK-4 protein, which is encoded by the IRAK-4 gene, receives signals from toll-like receptors and IL-1 receptor-related proteins. As a result of the stimulation of IL-1R, IL-18R, and many TLRs, which are first lines defense against infection, the myeloid differentiation primary response 88 (MyD88) protein is the first adaptor protein that is connected to the receptors. The accumulation of IRAK-4 proteins in the area of infection and the activation of IRAK-4 kinase activity follow this process (6).

Zhang et al. scanned SNPs in allergic rhinitis for the IRAK-4 gene and detected a relation between the rs3794262 variant and allergic rhinitis (7). Koziczak-Holbro et al. demonstrated that IRAK-4 was a key component in the development of proarthritis inflammation by using mice devoid of IRAK-4 kinase activity (8). We decided to examine IRAK-4 rs3794262 (Chr.12: 43771627, intron, A/T substitution, minor allele frequency = 0.27) and rs4251481(Chr.12: 43774562, intron, A/G substitution, minor allele frequency = 0.08) polymorphisms, which have been studied in a small number of studies.

In this study, we aimed to investigate whether IRAK-4 rs3794262 and rs4251481 polymorphisms predisposed to IBD and the possible effects of these polymorphisms by examining these gene polymorphisms using clinical and prognostic parameters of IBD.

## 2. Material and methods

### 2.1. Subjects

One hundred seven patients with IBD, including 63 with ulcerative colitis and 44 with Crohn’s disease, who were admitted to the Department of Gastroenterology between 2016 and 2017, were enrolled in the study. In each patient, two endoscopic biopsies were taken from different colonic locations during a colonoscopy examination. The inclusion criteria for the study groups were based on the European Crohn’s and Colitis Organization. The control group, however, consisted of 103 healthy individuals with a negative history of IBD. All participants gave written informed consent, and the local ethics committee (Ethics Committee of İstanbul University, İstanbul Faculty of Medicine, No: 1751/2015) review board approved the study protocol.

### 2.2. DNA Isolation 

Genomic DNA was extracted from blood samples using an Invitrogen PureLink genomic DNA kit .1

### 2.3. Determination of IRAK-4 SNPs

Genotyping analysis of IRAK-4 SNPs was performed using real-time polymerase chain reaction (RT-PCR). The reactions were conducted in final volumes of 20 μL per patient, containing 10 µL TaqMan® Genotyping Master Mix, 7.5 µL of dH2O, 1 µL of 20X genotyping assay, 0.5 µL of TaqMan® probe customized for genotyping of SNPs of IRAK-4 gene (for SNP ID rs3794262; C__28964290_10 and for SNP ID rs4251481; u2932). 

Amplification was performed using a fast real-time PCR system (Applied Biosystems 7500 Fast Instrument, Waltham, MA, USA) and an Applied Biosystems StepOnePlus™ instrument (Applied Biosystems, Waltham, MA, USA), in the following steps: pre-PCR, with a duration of 1 min at 60 °C; preincubation of the reaction mixture at 95 °C for 10 min; thermocycling at 95 °C for 15 s and 60 °C for 60 s for 40 cycles; and post-PCR, with a duration of 1 min at 60 °C. 

### 2.4. Statistical analysis

Categorical variables such as genotypes and alleles were compared using the chi-square (χ2) test. Allele and genotype frequencies were determined through direct counting. Whenever an expected cell value of less than 5 was obtained, Fisher’s exact test was used. Differences in continuous variables between carriers and the control subjects were tested using Student’s t-test. Statistical analyses were performed using the SPSS 21.0 software (SPSS Inc., Chicago, USA). 

## 3. Results

Clinical and demographic parameters are shown in Table 1. There were no statistical differences between the study groups according to age (P > 0.05). According to genotype and allele distribution, there was no relationship between the rs3794262 genotypes and study groups (P > 0.05). The frequency pattern of rs4251481 AG genotype (95% CI: [1.15–15.38]; P = 0.029) and G allele (95% CI: [1.52–19.38]; P = 0.005) in all patients was statistically significant compared with the control group. In addition, the rs4251481 AA genotype was observed at a significantly greater frequency in the control group than in all patients in the study (95% CI: [0.05–0.65]; P = 0.005). However, no association was found between IRAK-4 variants and the UC and CD groups (Table 2).

**Table 1:** Clinical and demographical parameters of the study groups.

Clinical parameters	All patients(n=107)	UC(n=63)	CD(n=44)	Controls (n=103)	P-value
Age (year)	43.77 ± 13.63	45.71 ± 14.90	40.98 ± 10.30	44.33 ± 13.88	>0.05
Sex (male/emale)	53/54	30/33	23/21	50/53	-
C-reactive protein (mg/L)(reference range 0–5 mg/L)	1.08 ± 2.82	1.03 ± 3.36	1.14 ± 1.85	0.49 ± 1.15	>0.05
Erythrocyte sedimentationrange (mm/h)	27.08 ± 20.27	27.34 ± 20.49	26.70 ± 20.19	28.80 ± 11.43	>0.05
Demographical parameters	UC(n=63)		CD(n=44)		
Disease extension in UC Proctitis (n/%)Left-sided colitis (n/%)Extensive colitis (n/%)	30/48.415/24.217/27.4		---		
Disease localization Ileal (n/%)Ileocolonic (n/%)Colonic (n/%)	---		20/45.520/45.54/9		
Disease behavior Inflammatory (n/%)Penetrating (n/%)Structuring (n/%)Penetrating and structuring (n/%)	----		21/48.88/18.613/30.21/ 2.3		

n = number of subjects, UC = ulcerative colitis, CD = Crohn’s disease.

**Table 2:** Distribution of the IRAK-4 rs4251481 and rs3794262 gene variants in the study groups.

IRAK-4 rs4251481	All patients(n=107)	UC(n=63)	CD(n=44)	Controls (n=103)
AA (n/%)	92/86	55/87.3	37/84.1	100/97.1*,***
GG (n/%)	3/2.8	2/3.2	1/2.3	-
AG (n/%)	12/11.5*	6/9.5	6/13.6**	3/2.9
A (n/%)	196/91.5	116/92.1	80/90.9	203/98.5
G (n/%)	18/8.5*	10/7.9***	8/9.1**	3/1.5
IRAK-4 rs3794262	All patients(n=107)	UC(n=63)	CD(n=44)	Controls (n=103)
TT (n/%)	80/74.8	47/74.6	33/75	81/78.6
AA (n/%)	2/1.9	2/3,2	-	-
TA (n/%)	25/23.4	14/22.2	11/25	22/21.4
T (n/%)	185/86.4	108/85.7	77/87.5	184/89.3
A (n/%)	29/13.6	18/14.3	11/12.5	22/10.7

n = number of subjects, UC = ulcerative colitis, CD = Crohn’s disease, * all patients vs control, for AG genotype P = 0.029; for G allele P = 0.005; for AA P = 0.005 ** CD vs control, for AG genotype P = 0.021; for G allele P = 0.008 *** UC vs control, for G allele P = 0.022; for AA P = 0.022.

Similar to the comparison between all patients and the control group, the frequencies of the rs4251481 AG genotype (95% CI: [1.25–22.11]; P = 0.021) and G allele (95% CI: [1.54–25.67]; P = 0.008) were greater in the CD group than in the control group. In the UC group, only the rs4251481 G allele was observed as a risk factor for disease (95% CI: [1.23–19.02]; P = 0.022). The frequency of the rs4251481 AA genotype was greater in the control group compared with the UC group (95% CI: [0.05–0.80]; P = 0.022) (Table 2).

No significant relationship was found between gene variants and clinical/demographic parameters in the patient groups. However, although C-reactive protein (CRP) levels were within the reference range (0–5 mg/L), patients with UC who carried the IRAK-4 rs4251481 GG genotype had increased CRP (AA = 1.10 ± 3.60; AG = 0.36 ± 0.26; GG = 1.25 ± 0.63) and erythrocyte sedimentation rates (mm/h)****(AA = 26.30 ± 20.19; AG = 33.00 ± 25.82; GG = 38.00 ± 1.14) compared with AG and AA, but the differences were not significant. 

## 4. Discussion

IBD is a chronic and complex genetic disease. Although the role of microbial factors, bacterial clearance defects, and immune response defects are known, the etiologies of IBD are not yet well understood (9).

IRAK-4 gene interacts with MyD88, which leads to the activation of nuclear factor-κb (NF-κB) and the transcription of inflammatory mediators. It was reported that in IRAK-4 knock-out rats that many TLR ligands and intracellular IL-1, IL-18 responses were extreme (10). Kondo et al. suggested that AS2444697, which is an IRAK-4 inhibitor, suppressed the progression of chronic renal failure via antiinflammatory action and might be potentially useful in treating patients with chronic kidney disease (11). 

As a result of a literature review, it was seen that there are a limited number of studies on IRAK-4 and IBD, which is an important pathway of this route. For this reason, the purpose of our study was to determine the efficiency of IRAK-4 gene variants, which has not previously been investigated in IBD.

In the present study, we found no association between IRAK-4 rs3794262 genotypes and the study groups. However, there was a remarkable difference regarding IRAK-4 rs4251481 genotypes between the study groups. The difference in frequency of the rs4251481 AG genotype (P = 0.029) and the G allele (P = 0.005) in all patients was statistically significant compared with the control group. Also, in the CD group, the frequencies of the rs4251481 AG genotype (P = 0.021) and the G allele (P=0.008) were greater than in the control group. In the UC group, only the rs4251481 G allele was observed as a risk factor for disease (P = 0.022). 

Zhang et al. reported that two SNPs (rs3794262, rs4251481) showed a strong association with allergic rhinitis in patients who were allergic to house dust mites, but not in patients allergic to pollens or mixed allergens (7). No functional effects of these IRAK-4 SNPs on the gene function such as inhibition or activation were observed. However, our results confirm this positive association between IRAK-4 genotypes and IBD as an inflammatory disease.

There were no statistically significant relationships between demographic parameters and IRAK-4 genotypes in the UC and CD groups (P > 0.05). Furthermore, we observed no differences between the patients and controls in terms of biochemical parameters. However, patients with UC who carried IRAK-4 rs4251481 GG genotype had increased CRP and sedimentation rates compared with AG and AA, albeit nonsignificantly. Our results are similar to our genotyping results, which showed that the G allele is a risk factor for UC and the AA genotype is a protective genotype against UC. Some studies showed that CRP and erythrocyte sedimentation rates were important for the diagnosis and determination of clinical activity of IBD (12–14). CRP may be a useful predictor of surgery in subgroups of patients with either UC or Crohn’s disease. It was demonstrated by Sachar et al. that the rate of increase in sedimentation range with progressively increasing clinical activity from mild to moderate was the same in all disease categories, with the exception of Crohn’s disease (13). Henriksen et al. indicated that CRP levels at diagnosis were related to the extent of disease in patients with UC (14).

There are some limitations of this study, one of which is the relatively small size of the study population. Another limitation is that we could not use any other techniques, such as ELISA for IRAK-4, to support our results.

To our knowledge, this is the first study to suggest that the association of the AG genotype and G allele of rs4251481 variants in the IRAK-4 gene with susceptibility to IBD. 

## Acknowledgment

This work was supported by the Scientific Research Projects Coordination Unit of İstanbul University. Project number: 59062
